# Scientific and Legal Perspectives on Science Generated for Regulatory Activities

**DOI:** 10.1289/ehp.9978

**Published:** 2007-11-07

**Authors:** Carol J. Henry, James W. Conrad

**Affiliations:** American Chemistry Council, Arlington, Virginia, USA

**Keywords:** Administrative Procedure Act, agency proceedings, conflict of interest, financial disclosure, industry science, Information Quality Act, interested persons, peer review, regulatory science, right to publish, scientific quality

## Abstract

This article originated from a conference that asked “Should scientific work conducted for purposes of advocacy before regulatory agencies or courts be judged by the same standards as science conducted for other purposes?” In the article, which focuses on the regulatory advocacy context, we argue that it can be and should be. First, we describe a set of standards and practices currently being used to judge the quality of scientific research and testing and explain how these standards and practices assist in judging the quality of research and testing regardless of why the work was conducted. These standards and practices include the federal Information Quality Act, federal Good Laboratory Practice standards, peer review, disclosure of funding sources, and transparency in research policies. The more that scientific information meets these standards and practices, the more likely it is to be of high quality, reliable, reproducible, and credible. We then explore legal issues that may be implicated in any effort to create special rules for science conducted specifically for a regulatory proceeding. Federal administrative law does not provide a basis for treating information in a given proceeding differently depending on its source or the reason for which it was generated. To the contrary, this law positively assures that interested persons have the right to offer their technical expertise toward the solution of regulatory problems. Any proposal to subject scientific information generated for the purpose of a regulatory proceeding to more demanding standards than other scientific information considered in that proceeding would clash with this law and would face significant administrative complexities. In a closely related example, the U.S. Environmental Protection Agency considered but abandoned a program to implement standards aimed at “external” information.

## Standards and Practices for Judging the Quality of Scientific Work

The project on Scientific Knowledge and Public Policy (SKAPP) examines the nature of science and the ways in which it is used and misused in government decision making and legal proceedings. Last year, SKAPP commissioned papers to address the question “Should scientific work conducted for purposes of advocacy before regulatory agencies or courts be judged by the same standards as science conducted for other purposes?” (SKAPP 2006). This article is adapted from one of those papers.

Science is a social enterprise, and scientific work tends to be accepted by the community when it has been confirmed. Crucially, experimental and theoretical results must be reproduced by others within the science community and the validity of the work established by replication (Wikipedia 2007a). However, such replication can take years, and what constitutes replication in a given case may also be disputable. Consequently, a variety of standards and practices have been established over the years for assessing the quality of scientific work (Barrow and Conrad 2006). These standards and practices apply to both “testing” (activities conducted pursuant to protocols, prescribed by regulatory agencies, that specify the content and characteristics of studies to be conducted to meet regulatory requirements, e.g., a standard bioassay for carcinogenesis) and “research” (studies that are hypothesis driven, addressing broad methodologic or mechanistic questions, e.g., the biologic activity of chemicals on the environment).

Both government and the scientific community outside of government have played independent but reinforcing roles in developing and propagating these standards and practices. The private sector is actually driving implementation of several relatively newer practices, in part to address concerns about the credibility of industry-funded science. In general, the regulatory implementations of these concepts impose additional requirements well beyond those conventionally imposed outside of the regulatory context. These government requirements promote a high degree of reliability. As explained below, the cumulative result of their applicability is that science conducted for regulatory purposes in many cases is actually likely to be more reliable than science conducted outside the regulatory arena. This is not to argue that the latter should be required to comply with these government requirements to any greater extent than it already is, but only to emphasize that science conducted pursuant to them is relatively more reliable as a result.

### Information Quality Act

The Information Quality Act (IQA 2000) governs the quality of information that federal agencies disseminate. The IQA required the Office of Management and Budget (OMB) to issue an initial set of implementing guidelines (OMB 2002). Each federal agency then issued its guidelines applying the OMB guidelines to its particular circumstances [e.g., U.S. Environmental Protection Agency (U.S. EPA) 2002a].

It is important to note that the IQA applies not only to information that agencies generate themselves but also to information developed by nongovernment parties, to the extent the government “disseminates” it, either by adopting or endorsing it as its own view or by relying on it to make a decision. [Information prepared for administrative adjudications is exempt from IQA guidelines, but agencies construe this exemption narrowly (U.S. EPA 2002a).] Thus, to the extent that businesses, universities, or other private entities conduct research or testing and the results come into the possession of an agency such as the U.S. EPA, the results cannot form a basis of the agency’s decision without becoming subject to IQA requirements. And those requirements are most precise and demanding in the area of scientific information.

OMB’s guidelines (OMB 2002) prescribe fairly detailed standards for “objectivity.” As a general matter, information must be accurate, reliable, and unbiased. Scientific information must be generated using sound research methods. The sources of the information must be disclosed and data should be documented. Scientific information must be accompanied by supporting data and models. “Influential” scientific information must be sufficiently transparent to be reproduced subject to several caveats. (“Influential” information is that which an agency “reasonably can determine will have or does have a clear and substantial impact on important public policies or private sector decisions.”) Influential information regarding risks to health, safety, or the environment must also be based on “the best available, peer-reviewed science and supporting studies conducted in accordance with sound and objective scientific practices; and . . . data collected by accepted methods or best available methods,” and must disclose significant uncertainties and relevant peer-reviewed studies.

Accordingly, if a business submitted a paper to the U.S. EPA regarding research it had conducted on the risks posed by one of its products, and the U.S. EPA was going to consider this paper as part of making a decision involving that family of products—a decision that would have a substantial impact on producers and customers of those products—that paper would be subject to the most demanding level of IQA objectivity standards. The underlying data and methods would have to be provided to the agency. Also, the agency (and hence the submitter) would have to be able to document that the research reported in the paper was conducted in accordance with sound and objective scientific practices. Finally, the paper would need to be based on the best available science, including any peer-reviewed work in the literature. [Notably, by virtue of being provided to a federal agency, this information would become subject to the Freedom of Information Act (FOIA 1966). Exemptions to FOIA exist for privacy and commercial considerations, although health effects data generally cannot be claimed as confidential business information (Conrad 2006).]

### Good Laboratory Practice regulations

Both the U.S. EPA and Food and Drug Administration (FDA) have adopted comparable sets of requirements specifying laboratory practices and procedures that must be followed to ensure the quality and integrity of studies submitted to support agency decisions (FDA 2006; U.S. EPA 2006a, 2006b). These Good Laboratory Practice (GLP) standards prescribe essential, routine features of sound laboratory science. All studies submitted to these agencies in connection with the relevant statutory programs must be conducted in accordance with these standards. The GLP rules are more rigorous than standards followed at university laboratories (Anderson et al. 2001).

GLPs have three basic elements: quality assurance, standard operating procedures, and study protocols.

#### Quality assurance

GLPs mandate documentation of study conduct and results and ensure that a full record of the study is preserved for subsequent review, if necessary.

#### Standard operating procedures

There must be written procedures for accurate and full data collection under the nonclinical laboratory study methods to be used, methods determined by management to be adequate to ensure the quality and integrity of the data.

#### Study protocols

Each study must state the objectives and all methods for the conduct of the study in a clearly written protocol that must be approved by the agency. These protocols have been validated and chosen, after extensive and careful review, to provide an acceptable degree of scientific certainty, in the agency’s view, regarding the reliability and relevance of test results, which in turn provides the confidence necessary for making safety determinations and other regulatory determinations.

Conformance to GLPs does not, in itself, ensure that scientific work is reproducible, or that the resulting data will be interpreted correctly. However, when research studies adhere to GLPs, reviewers and those acting upon the science may have a high degree of confidence that the experimenters *a*) adhered to the experimental protocol employed, *b*) took all the steps and measurements claimed to be taken during conduct of the study itself, and *c*) accurately reported the test results.

GLPs are neither required nor common in research laboratories but have been implemented at all U.S. EPA contract laboratories and at major universities that perform significant medical and toxicology testing and research for regulatory purposes. Sponsoring organizations can require that research and testing studies comply with GLPs and can incorporate that request into contractual vehicles. As a result, GLPs have grown to be used fairly extensively outside the specific U.S. EPA and FDA regulatory contexts in which they are required.

### Peer review

While peer review has been an integral part of medicine for centuries, it has become a mainstay of the scientific process only since the mid-twentieth century (Wikipedia 2007b). The National Research Council (NRC) has defined peer review as providing an “in-depth critique of assumptions, calculations, extrapolations, alternate interpretations, methodology, and acceptance criteria employed and conclusions drawn in the original work” (NRC 1998). At a minimum, peer review must exhibit the following features (International Life Sciences Institute 2005):

- It must include multiple assessments.
- It must be conducted by scientists with no direct connection to the research, or its sponsors.
- It must be conducted by scientists who have experience with or expertise in the research in question.

A rigorous peer review is a key part of the foundation on which scientific excellence is achieved in all research programs. Science is not “self-evident” and requires scientific judgment, and because judgments vary, a well-balanced representation of intellectual perspectives is needed. Where that is obtained, the peer review is more likely to rebut any scientifically untoward or untenable hypothesis and arrive at a successful review.

#### The crucial role of reviewer independence and expertise

The hallmark of any peer review is the independence and expertise of the peer reviewer. Reviewer knowledge, experience, and expertise are central requirements for instructive peer review and ensure “technical credibility” for the review. The peer reviewer should have expertise at least equivalent to that needed for the “original work” but must be independent of the work being reviewed. These dual requirements for expertise and independence offer the best chance of obtaining objective, expert evaluation while maintaining scientific integrity (NRC 2003).

#### Conflict of interest and bias in peer review

Identifying and managing potential conflicts of interest (COIs)—financial and non-financial—is critical to meeting the requirement for independent peer review. In turn, this requires distinguishing between COI and bias.

The National Academies (2003) defines COI as any financial or other interest that conflicts with the service of an individual because it could impair the individual’s objectivity or create an unfair competitive advantage for any individual or organization.

Conventionally speaking, some COIs are “actual,” with an unambiguous potential for financial gain (e.g., the reviewer holds stock in an entity likely to be affected by the relevant regulatory action). Other actual COIs might be employment conflicts or close professional financial relationships. Potential financial COIs may exist on the part of experts remunerated by any individual or organization (International Life Sciences Institute 2005). Similarly, any scientist can have a COI, depending on the topic. As the Government Accountability Office (GAO 2001) has noted, association with industry does not by itself indicate a COI. Conversely, association with an environmental group does not inoculate a scientist against COIs. There well may be COIs among academics, because of intense competition for research funds and publications, that are difficult to identify.

An individual with a COI generally may not participate in a peer review. Organizations that use peer-review systems require candidates to disclose their organizational affiliations, financial interests, personal and professional involvement, and other information that may be pertinent to the topic, erring on the side of full disclosure, and to certify that the information is true and accurate to the best of their knowledge.

“Bias” is a partiality or loss of objectivity because of personal views or positions and may be perceived as arising from the close identification or association of an individual with a particular point of view or with a particular group that may be affected by the research being reviewed. For example, questions about the neutrality of a reviewer could arise if that person had represented an interest group at a hearing.

It is generally recognized that bias is pervasive and not inherently undesirable. A subcommittee of the U.S. EPA Science Advisory Board (2000) has opined that, “[a]lthough it is possible to avoid conflict of interest, avoidance of bias is probably not possible. All scientists carry bias due, for example, to discipline, affiliation and experience.” Potential sources of bias in peer reviewers are managed through disclosure.

Federal regulations generally implement these concepts. Although these rules address only federal employees, they include “special government employees,” such as participants in panels organized under the Federal Advisory Committee Act (1972), and therefore they apply in the case of federal peer review panels such as the U.S. EPA Science Advisory Board. These rules prohibit a federal employee from participating directly and substantially in a particular matter (as opposed to “broad policy options”) that will have a direct and predictable effect on *a*) a financial interest of the employee (generally, employment or stock ownership); *b*) the employee’s employer; or *c*) organizations for which the person has served in the last year in a paid capacity or as an active participant, where a reasonable person would question the person’s impartiality in the matter, unless covered by an exclusion or issued a waiver (Office of Government Ethics 1997).

#### Peer review at federal agencies

Historically, the primary venues for peer review have been in journal publication and in grant evaluation. Increasingly, federal agencies have been conducting more demanding peer reviews of studies that will form the basis of important agency decisions. For example, the U.S. EPA has several standing advisory bodies to conduct peer reviews, including its Science Advisory Board, its Science Advisory Panel (for pesticide program decisions), and its Clean Air Science Advisory Committee. Some agencies request the National Research Council of the National Academies to convene peer reviews for very prominent issues and assessments. Although the principles for journal and agency peer review are the same, they involve very different practices, procedures, and decision pathways from the results and conclusions of the peer review.

Agency peer reviews are complex, time consuming, and most often involve forming a panel of expert peer reviewers, where the panel will meet in public for their deliberations. In some cases, peer reviews are conducted by scientists within the agency, and in other cases by external scientists. Prior criticisms of agency peer reviews (GAO 2001) have only heightened the scrutiny applied to them both within and outside agencies.

#### OMB’s peer review bulletin

Many federal agencies have written policies for when and how to conduct agency peer reviews (U.S. EPA Science Policy Council 2000). To bring greater consistency to and establish minimum requirements for such reviews, the OMB and the Office of Science and Technology Policy in early 2005 issued their “Final Information Quality Bulletin for Peer Review” (OMB 2005). The bulletin requires all federal agencies to conduct peer reviews of all “influential” scientific information—defined as under the IQA—that the agency intends to disseminate. [As with the IQA, information disseminated as part of an adjudication (e.g., a permit decision) is exempt from rules outlined in the bulletin, unless it is novel or precedential and peer review is practical.]

The bulletin sets especially high standards for “highly influential” scientific assessments. For these documents, *a*) agency scientists generally may not participate; *b*) reviewers must be provided with background information sufficient to understand the key findings or conclusions of the draft assessment; *c*) where feasible, public comment should be sought and provided to the reviewers; and *d*) the agency should respond to the reviewers’ report.

Under the guidelines of the OMB bulletin, peer review should now be applied systematically to all influential scientific information published by federal agencies or used by them to make decisions—regardless of why the work was conducted—in a manner that is more rigorous and revealing than that occurring outside the federal government.

### Disclosure and acknowledgment of funding sources

In the mid-1990s, concern arose about the integrity of scientific research because of increasing commercial links and consequent influences. In response, the Department of Health and Human Services (DHHS) and the National Science Foundation (NSF) issued their “Investigator Financial Disclosure Policy” (DHHS/NSF 1995). This policy required disclosure of an investigator’s significant financial interest if it “would reasonably appear to be affected” by activities funded or proposed for funding by NSF and DHHS.

As the scientific community became more aware of the potential COIs for university investigators with commercial ties receiving federal grant support, questions also emerged about the potential implications for interpretation of research results and conclusions. By 2001 some of the major scientific journals had established policies to encourage authors to declare any competing financial interests in relation to research papers. Financial disclosure forms are now routinely required to be submitted with manuscripts for use by the editors, and journal articles generally acknowledge research support, although the practice is not universal (Nature 2006; Science 2006).

Although journals have instituted disclosure policies (sometimes termed “competing financial interests policies”) to increase transparency for their readers, granting agencies and sponsors employ a variety of approaches and requirements, and it is too early to conclude that a broad consensus practice has been established. The Long-Range Initiative (LRI) of the American Chemistry Council (ACC) requires its contractors to acknowledge ACC as a sponsor of the research in all articles or publications pertaining to the research conducted under agreement with the ACC.

### Transparent research policies

Research programs are commonly regarded as more credible, and their results less suspect, when they employ transparent processes and procedures regarding ownership of data, release of results, and publication of results. This practice is growing for assessing scientific quality, although it has not yet achieved broad acceptance within the research community.

When investigators own the data and the scientific information that they generate through their research efforts, they are in control of how those data will be evaluated, used, and communicated. Sponsors who choose to employ this approach do so to lend strength, objectivity, and credibility to the outcome of the research. The ACC LRI program includes this element of data ownership (ACC 2006).

With ownership of the data—the right to release data independently and to publish without prior sponsor approval—inappropriate sponsor interference can be avoided. Interactions with sponsors can be beneficial in providing specialized knowledge and insight at a level of detail other scientific resources might not provide. However, the final decision on whether to accept the sponsor’s information or advice remains with the investigator.

Congress has required the federal government to take a slightly different tack in the area of research transparency. As a result of the “Shelby Amendment” (Omnibus Appropriations Act 1998), the OMB revised its Circular A-110 governing federal grants, and now all federal agencies must make available to the public, pursuant to FOIA, final research data generated by agency grantees that an agency cites in support of a rule or order (OMB 1999). Thus, when the federal government funds scientific work and relies on it to support agency action with the force and effect of law, the data produced by that work will be placed in the public domain if someone in the public asks for it. This way, federally funded research data can be available so that the scientific community can validate the work through replication. Although Circular A-110 does not apply to privately funded research submitted to federal agencies, the same prospect of disclosure is an inevitable effect of the IQA, discussed above, if the agency relies on that information.

## Federal Standards for Considering “Regulatory” Science

The foregoing discussion outlines the standards that one can use to judge the quality of scientific research without regard to the purpose for which it was conducted. The following discussion explains why regulatory agencies should not—and arguably cannot—treat science created for purposes of an agency proceeding differently in that proceeding than science not created for purposes of that proceeding.

### The breadth of research and testing obligations

The data proffered by regulated parties in agency proceedings often are not solely the results of self-interested efforts to influence those proceedings. Rather, in many cases the parties have been obligated by regulation or order to conduct particular studies, according to particular protocols, and to provide the results of that work to the agency (Conrad 2006).

For example, under Section 4 of the Toxic Substances Control Act (TSCA 1976), the U.S. EPA has broad power to issue rules ordering persons manufacturing, processing, or importing a chemical to conduct further tests regarding the chemical’s health or environmental effects. TSCA Section 8(d) authorizes the U.S. EPA to compel, by rule, manufacturers and importers of a given chemical to submit lists and copies of existing, unpublished health and safety studies for the chemical. TSCA mandates have resulted in “more than 50,000 studies covering a broad range of health and ecological endpoints” being filed with the U.S. EPA since 1976 (U.S. EPA 2003a).

Similarly, the Federal Insecticide, Fungicide, and Rodenticide Act (1972) requires any potential pesticide chemical to undergo more than 100 scientific tests addressing chemistry, health effects, environmental effects, and residue chemistry to determine whether it can be used safely (U.S. EPA 2006c). Only after the information has undergone a thorough and rigorous review by the U.S. EPA can the product be “registered” by the U.S. EPA for use to protect crops or public health.

Most federal processes for evaluating the safety of chemicals, including the FDA review of drug applications (Federal Food, Drug, and Cosmetic Act 1938), depend heavily on privately generated data. Indeed, historically and for the foreseeable future, the vast majority of chemical and product testing has been and will be borne by industry, not the public sector. Policies regarding the treatment of the resulting data need to bear this reality in mind.

### The rights of interested persons under federal administrative law

The notion that science generated for regulatory purposes should be evaluated differently than other science is premised on the idea that the self-interest of regulated parties that conduct or sponsor research creates a conflict with the interest of the truth, whether conscious or unconscious, that renders the research invalid or at least suspect (Krimsky 2005).

At the outset, we acknowledge the growing literature purporting to find that industry-funded research produces results that favor its sponsors more often than other research on the same topic (Krimsky 2005; vom Saal and Hughes 2005). As the authors of one of those studies has fairly observed in comparing industry and government-funded studies, one or both of two things could be going on: either “industry-funded scientists [are] under real or perceived pressure to find and publish only data suggesting negative outcomes” or “government-funded scientists [are] under real or perceived pressure to publish only data suggesting adverse outcomes” (vom Saal and Hughes 2005). Indeed, the source of a scientist’s funding may be less a cause of bias than an effect of it. As an editor of *The Lancet* (Horton 1997) has argued, financial conflicts “may not be [more] influential” than underlying biases, because “interpretations of scientific data will always be refracted through the experiences and biases of the authors.” Similarly, a student of the science/policy interface (Sarewitz 2004) has argued that

stripping out conflicts of interest and ideological commitments to look at ‘what the science is really telling us’ can be a meaningless exercise [because] even the most apparently apolitical, disinterested scientist may, by virtue of disciplinary orientation, view the world in a way that is more amenable to some value systems than others.

As vom Saal and Hughes argued in their article (2005), the appropriate technical response when confronted with science conducted by interested parties is to use that fact as an alert to look, perhaps more deeply than one otherwise might have, for “what specific factors, other than source of funding,” may be associated with the results of that science.

Such an approach is clearly the only proper one in the case of interested research submitted to federal regulatory agencies, simply because the concept of “conflict of interest” is not employed in federal laws governing the regulatory process (aside from the government ethics rules noted above). Indeed, no federal laws, rules, or policies express a presumption that agencies in a given proceeding should ignore or give less weight to scientific work on the basis of who conducted or funded it or, more to the point, whether it was prepared specifically for the relevant proceeding. To the contrary, federal administrative law, as interpreted by the courts, generally evinces a congressional mandate that agencies give interested or affected parties access to and input into administrative processes. In effect, Congress and the courts have determined that in an open democratic society administered by a bureaucracy required to act fairly and rationally, it is important that agencies allow interested or affected persons to provide information to them, and fairly consider that information.

The backbone of federal administrative law is the Administrative Procedure Act (APA 1946), which requires agencies to provide notice and “give interested persons an opportunity to participate in [a] rulemaking through the submission of written data, views, or arguments. . . .” Courts interpreting Section 553 of the APA have made clear that an agency must consider and respond to—rather than discount—all significant matters put before it. In particular, courts have specifically rejected the notion that the APA should provide some basis for insulating agency officials from the input of regulated parties. In the words of former D.C. Circuit Judge Patricia Wald (*Sierra Club v. Costle* 1981):

Under our system of government, the very legitimacy of general policymaking performed by unelected administrators depends in no small part upon the openness, accessibility, and amenability of these officials to the needs of the public from which their ultimate authority derives, and upon whom their commands must fall. . . . Furthermore, the importance to effective regulation of continuing contact with a regulated industry, other affected groups, and the public cannot be underestimated. Informal contacts can . . . spur the provision of information which the agency needs.

In addition to setting a single set of quality standards for federally disseminated information regardless of provenance, the IQA also authorizes “affected persons to seek and obtain correction of information . . . disseminated by [a federal] agency that does not comply with the guidelines issued by [OMB].” Far from having their own information judged weighted less under the IQA, affected persons are empowered to use their own information to obtain correction of government information that does not meet the common set of standards that should apply to any information disseminated by the government.

Several other administrative law statutes embody the same orientation toward interested persons and their rights to submit information to federal agencies and have it considered. These include the Regulatory Flexibility Act (1980), the Paperwork Reduction Act (1980), and the Federal Advisory Committee Act (1972). The latter also requires advisory committees to be “balanced,” which should equally prohibit exclusion of, as well as domination by, any interest.

The upshot of this authority is not that regulated agencies are bound to accept unquestioningly any information that is generated specifically for that proceeding. Agencies can and, indeed, must assess the validity of information upon which they rely. But they cannot adopt blanket approaches that judge science generated for a regulatory proceeding differently than other science considered in that proceeding.

### What sorts of proceedings and entities would be covered

The next hurdle in imagining a system that imposed different standards on science created specifically for a given regulatory proceeding is to consider what sorts of agency “proceedings,” and what sorts of entities conducting or sponsoring science, would be covered. These turn out not to be simple determinations in all cases.

Where testing is being conducted by a manufacturer of a chemical or product (e.g., an exposure study conducted in support of a pesticide’s reregistration), there is little question that the research is being conducted for those proceedings. But manufacturers frequently conduct or sponsor research and testing for product stewardship and other business reasons. In some cases, that work may also be useful in some other agency “proceeding” (e.g., establishing a “reference concentration” value in the U.S. EPA Integrated Risk Information System database (http://www.epa.gov/iris). Such a proceeding may be ongoing, or the company may know the agency is contemplating it, or the company may plan to propose that the agency initiate it. What would the rules be in such “mixed-motive” cases? Conversely, such an agency proceeding may arise later and be truly unanticipated by the regulated entity. What sort of evidentiary process would have to be established to determine what the research proponent intended or knew at the time research was initiated? All these circumstances would need to be addressed in a system that tried to treat “regulatory proceeding” science differently.

Moreover, many regulatory settings are more than bilateral; that is, entities other than the agency and a single regulated party may be able to submit scientific information. Even if one accepted the premise that science prepared for purposes of a proceeding should be treated differently than other science, there is no inherent reason that science prepared for a proceeding by opponents of the permit should be treated differently than science prepared by its proponents. Indeed, many regulatory proceedings have multiple parties aligned with and against the agency and other parties on different issues in complex ways, making it difficult in many cases to determine who is on anyone’s “side.”

Finally, it will often be difficult to demarcate “nonregulatory” science, given the extent to which academic scientists are participants or are at least partisans in regulatory disputes. Although highly controversial issues generally raise important intellectual questions (e.g., the effects of pollutants at low doses), there is also no question that many of the academics working on these issues are highly invested both in their hypotheses and in the regulatory uses of their findings. Although these scientists may not have a financial or other tangible stake in any particular regulatory proceeding, it seems artificial and formalistic to say that their research is not being conducted, at least in part, so that its results can be used in regulatory proceedings.

Thus, any effort to establish special rules for consideration of science generated for regulatory proceedings will face difficult definitional challenges regarding what is a “proceeding,” even more difficult evidentiary challenges determining whether and the extent to which scientific work was being conducted for such proceedings, and politically loaded challenges over when “unaffiliated” or “academic” work was in fact being conducted, at least in part, for regulatory purposes.

### Case study: the U.S. EPA assessment factors for external information

The issues raised above ultimately led the U.S. EPA to abandon a related effort: to establish guidelines that treated “external” information differently than information whose generation the U.S. EPA controlled.

Early on, the U.S. EPA realized that the IQA would apply to information generated by third parties that the agency relied upon or otherwise disseminated. The U.S. EPA draft “Assessment Factors for Evaluating the Quality of Information from External Sources” (U.S. EPA 2002b) noted that

the Agency . . . receives information that is voluntarily submitted to EPA by external sources (‘third parties’) in hopes of influencing Agency actions. . . . The purpose of this document is to describe sets of ‘assessment factors’ that illustrate the types of considerations that EPA takes into account when evaluating the quality and relevance of information that is voluntarily submitted or that we obtain from external sources in support of various Agency actions.

The balance of the document consisted of an elaboration on five “categories of general assessment factors”: soundness, applicability and utility, clarity and completeness, uncertainty and variability, and evaluation and review.

Critics argued that there is no basis, under the IQA or any other legal authority, or indeed, on any technical grounds, for the U.S. EPA to assess “external” or “third-party” information by different standards than first-or second-party information. On technical grounds, critics noted that information generated by the U.S. EPA or its contractors is not immune from the same types of errors associated with information from external sources. For example, the U.S. EPA Inspector General had just issued a memorandum noting that the agency faced a number of unresolved challenges in “establishing quality assurance practices to improve the reliability, accuracy, and scientific basis of environmental data” (U.S. EPA Inspector General 2002). The Inspector General’s memo expressed similar concerns with respect to the accuracy and reliability of information generated by U.S. EPA contractors. Critics argued that there was ample justification for the agency to apply its proposed assessment factors to that information, as well as to information submitted by third parties.

Critics of the U.S. EPA draft assessment factors also argued that assessment factors for external information created the undesirable appearance of a double standard and opened the door to arbitrary agency decisions to exclude otherwise appropriate information received from external sources on the basis of the selective application of assessment factors to information products. Most important, they contended that the standards the agency offered for judging the quality and reliability of third-party data were no different than those that should be applied to evaluate information generated by the agency itself, U.S. EPA contractors, or U.S. EPA permittees, and hence a single set of assessment factors should apply to all.

When the U.S. EPA finalized the assessment factors document, it clarified that “the document does not constitute a new standard for information quality, nor does it describe a new process for evaluating third party information.” Second, and more important, it added that, “in general, we agree that consistent standards of quality should apply to both internally and externally generated information, when used for the same purposes” (U.S. EPA 2003b).

## Conclusion

Only one set of standards and practices should be used to judge the quality of scientific work in a given regulatory proceeding, regardless of why the work was conducted. It may be that, over time, more of these practices and standards will apply to all scientific information. Many of these hallmarks of scientific quality are incorporated into federal law, rules, and policy. These same federal authorities impose additional standards that further ensure the quality of scientific work generated or submitted for regulatory purposes. Federal laws also ensure that interested parties have a right to submit information for regulatory proceedings and to have that information considered fairly and on its merits. Any system of differential treatment for regulatory science would face severe scrutiny in light of that authority and would be difficult to administer. Most important, it would not necessarily lead to an improved scientific foundation for regulations.
